# AMPK suppresses Th2 cell responses by repressing mTORC2

**DOI:** 10.1038/s12276-022-00832-x

**Published:** 2022-08-23

**Authors:** Mahesh Pandit, Maheshwor Timilshina, Ye Gu, Suman Acharya, Yeonseok Chung, Sang-Uk Seo, Jae-Hoon Chang

**Affiliations:** 1 College of Pharmacy, Yeungnam University, Gyeongsan, 38541 Republic of Korea; 2 Laboratory of Immune Regulation, Research Institute of Pharmaceutical Sciences, College of Pharmacy, Seoul National University, Seoul, 08826 Republic of Korea; 3 Department of Microbiology, College of Medicine, The Catholic University of Korea, Seoul, 06591 Republic of Korea

**Keywords:** Lymphocytes, Inflammation

## Abstract

Allergic inflammation is a T helper 2 (Th2) cell-driven pathophysiological phenomenon, but the mechanism by which the metabolic cascade affects Th2 cell differentiation remains unclear. In this study, we investigated the roles of AMP-activated protein kinase (AMPK) and intracellular energy sensors in Th2 cell differentiation and the pathogenesis of allergic inflammation. Accordingly, T-cell-specific *AMPK* or Sirtuin 1 (*Sirt1*)-knockout mice were subjected to allergic inflammation, and their Th2 cell responses were investigated. The results demonstrated that inducing allergic inflammation in *AMPK*- and *Sirt1*-knockout mice increased Th2 cell responses and exacerbated allergic phenotypes. Furthermore, treatment with 5-aminoimidazole-4-carboxamide ribonucleotide (AICAR), an activator of AMPK, ameliorated allergic inflammation in mice. Mechanistically, our findings revealed that AMPK repressed mechanistic target of rapamycin complex 2 (mTORC2), which downregulated the expression of suppressor of cytokine signaling 5 (SOCS5) in CD4^+^ T cells. In addition, the loss of AMPK signaling reduced SOCS5 expression and increased interleukin-4-STAT6–GATA3 axis-mediated Th2 cell differentiation. Finally, the T-cell-specific deletion of *Rictor*, a member of mTORC2, in *Sirt1*^T-KO^ mice led to the reversal of allergic exacerbation to the level in control mice. Overall, our findings suggest that AMPK in CD4^+^ T cells inhibits the differentiation of Th2 cells by repressing mTORC2 and thus serves as a potential target for Th2 cell-associated diseases.

## Allergic inflammation: When T-helper cells aren’t helping

Boosting activity of an energy-regulating enzyme helps reduce the severity of allergic inflammation driven by T-helper 2 (Th2) immune system cells. In allergic inflammation, the body overreacts to an allergen, and too many Th2 cells are activated. Although some of the molecules involved are known, the details are not well understood. Researchers from South Korea led by Jae-Hoon Chang at Yeungnam University, Gyeongsan, and Sang-Uk Seo at The Catholic University of Korea, Seoul, investigated how the AMPK enzyme, known to regulate T cells in response to cellular energy levels, affects Th2 cell differentiation. Mice lacking AMPK had increased numbers of Th2 cells and prone to allergic inflammation. Administering a drug that boosts AMPK fully alleviated allergy symptoms in mice. Improving our understanding of how Th2 cells are activated may help in finding better treatments for allergic inflammation.

## Introduction

In response to antigen-specific activation, multiple subsets of T helper (Th) cells are generated from naive ancestor cells to meet the requirements of the immune system^1^. Depending on the direction of Th-cell differentiation, T cells secrete specific sets of cytokines and consequently shape disease phenotype. Th2 cells are major players in the immune response to helminths and are implicated in inflammatory diseases associated with allergies. T-cell receptor (TCR) stimulation triggers Th2 differentiation through signal transducer and activator 6 (STAT6) in the presence of interleukin (IL)-4. STAT6 has been shown to promote the expression of the Th2 master regulator GATA-binding protein 3 (GATA3), and STAT6-deficient mice have been reported to be incapable of generating Th2 cell responses^2,3^. In most cells, the suppressor of cytokine signaling (SOCS) protein family inhibits IL-4 signaling and downregulates Th2 cell differentiation^4,5^. Furthermore, Th2 cell differentiation involves extensive metabolic reprogramming, and mammalian target of rapamycin (mTOR) plays a notable role in T-cell differentiation. mTOR complex 2 (mTORC2), which is a multiprotein complex, has been reported to be involved in the regulation of Th2 differentiation by inhibiting SOCS protein expression^5,6^. Although the molecular mechanism by which mTORC2 regulates Th2 differentiation has been extensively studied, the upstream metabolic regulation of Th2 cell differentiation remains unclear.

AMPK is a serine/threonine protein kinase with a trimeric structure consisting of a catalytic subunit (α subunit) and two regulatory subunits (β and γ subunits) that functions as a key metabolic regulator for a variety of physiological processes, such as cellular energy balance, substrate metabolism, protein synthesis, and cell proliferation^7^. A previous study showed that AMPK regulates T-cell effector functions in response to glucose availability^8^. Metformin, which is known to activate AMPK signaling, ameliorates the clinical severity of acute graft-versus-host disease and is associated with reduction in the numbers of Th1 and Th17 cells and increases in Th2 and Treg cells^9^. Moreover, the attenuation of AMPK by ROQUIN, a posttranscriptional repressor of T cells, promotes T follicular helper (Tfh) cell formation^10^. While CD4^+^ T cells that are deficient in liver kinase B 1 (LKB1), a known upstream regulator of AMPK, have been reported to have increased T-cell activation and inflammatory cytokine production, AMPK-deficient CD4^+^ T cells do not show any altered phenotype^11,12^. In addition, the Treg-specific ablation of *Lkb1 gene* resulted in increased inflammation in mice, but the deletion of AMPK in Tregs did not result in inflammatory phenotypes^13,14^. These results suggest that AMPK participates in T-cell differentiation independent of LKB1. However, the involvement of AMPK in CD4^+^ T-cell biology remains unclear.

In this study, we investigated the effect of T-cell-specific AMPK-knockout on the susceptibility of mice to allergic inflammation. In addition, an AMPK activator alleviated the severity of allergic inflammation in mice. We suggest that AMPK is a potential therapeutic target to control Th2-mediated inflammatory diseases.

## Materials and methods

### Mice

Wild-type, *Prkaa1*^fl/fl^, *Sirt1*^fl/fl^, *Rictor*^fl/fl^, and CD4-Cre transgenic C57BL/6 mice were purchased from the Jackson Laboratory (ME, USA) and inbred at the Yeungnam University Animal Center (Gyeongsan, Republic of Korea). Eight-week-old female BALB/c mice were purchased from Samtako Bio Korea (Gyeonggi, Republic of Korea). Floxed mice were crossed with CD4-Cre mice to generate *Prkaa1*^fl/fl^CD4-Cre (*Prkaa1*^T-KO^), *Sirt1*^fl/fl^CD4-Cre (*Sirt1*^T-KO^), *Rictor*^fl/fl^CD4-Cre (*Rictor*^T-KO^), and *Sirt1*^fl/fl^*Rictor*^flfl^CD4-Cre double-knockout (DKO) mice. The mice were studied at 8–10 weeks of age. Age- and sex-matched CD4-Cre mice (hereafter referred to as WT) were used as the control. All mice were housed in a specific pathogen-free facility at the Animal Center of Yeungnam University. Animal experimental protocols were approved by the Institutional Animal Care and Use Committee of Yeungnam University (Approval Number: 2014-018 and 2017-034).

### CD4^+^ T-cell isolation and culture

CD4^+^ T cells were isolated from single-cell suspensions of the spleen and lymph nodes using CD4-conjugated magnetic beads (Miltenyi Biotech, Bergisch Gladbach, Germany). Naive CD4^+^ T cells were sorted using a BD FACSJazz™ cell sorter (BD Biosciences, also known as Becton, Dickinson and Company, NJ, USA). Sorted naive T cells were cultured in RPMI supplemented with 10% FBS and 1% penicillin–streptomycin. Naive CD4^+^ T cells were activated with plate-bound anti-CD3 (5 μg/mL; Cat #100359; BioLegend, CA, USA) and soluble anti-CD28 (1 μg/mL; Cat # BE0015-1; Bio X Cell, NH, USA). For in vitro differentiation, the following cytokines were added: for Th1 differentiation, IL-12 (10 ng/mL) and anti-IL-4 (5 μg/mL) were added; for Th17 differentiation, TGF-β (2 ng/mL), IL-6 (10 ng/mL), anti-IL-4 (5 μg/mL), and anti-IFN-γ (5 μg/mL) were added; and for Th2 differentiation, IL-4 (10 ng/mL), IL-2 (10 ng/mL), and anti-IFN-γ (5 μg/mL) were added.

### Flow cytometry

Single-cell suspensions were prepared from the spleen and lymph nodes. The cells were incubated at room temperature for 2 min with red blood cell lysis solution (Millipore Sigma, MO, USA), washed with RPMI, and suspended in RPMI supplemented with 10% FBS and 1% antibiotics. To analyze surface markers, the cells were stained with the following fluorescence-conjugated antibodies at 4 °C for 15 min: FITC-conjugated anti-CD3ɛ (Cat # 145-2C11), PerCP-Cyanine5.5-conjugated anti-CD4 (Cat # GK1.5), PE/Cyanine7-conjugated anti-CD8 (Cat # 53-6.7), PE-conjugated anti-CD25 (Cat # PC-61), APC-conjugated anti-CD44 (Cat # IM7), PE/Cyanine7-conjugated anti-CD62L (Cat # MEL-14), APC-conjugated anti-CD71 (Cat # R17217), PE-conjugated anti-IFN-γ (Cat # XMG1.2), APC-conjugated anti-IL-17 (Cat # TC11-18H10.1), APC-conjugated anti-IL-4 (Cat # 11B11), APC-conjugated anti-IL-5 (Cat # TRFK5), PE-conjugated anti-CD98 (Cat # RL388), PE-conjugated anti-GATA3 (Cat # 16E10A23), and PE-conjugated anti-T-bet (Cat # 4B10) from BioLegend; APC-conjugated anti-Foxp3 (Cat # FJK-16s) and PE-conjugated anti-IL-13 (Cat # eBio13A) from eBioscience; and PE-conjugated anti-RORγt (Cat # Q31-378) from BD Biosciences. For intracellular cytokine staining, the cells were stimulated for 4–6 h with PMA (50 ng/mL; Cat # P8139, Millipore Sigma) and ionomycin (750 ng/mL; Cat # I9657, Millipore Sigma) in the presence of GolgiStop (10 μg/mL; Cat # 554724, BD Biosciences), and the fixed cells were incubated with the appropriate antibodies for analysis. FoxP3 staining was performed using anti-Foxp3 (Cat # FJK-16s; eBioscience, Thermo Fisher Scientific Corp, MA, USA) according to the manufacturer’s instructions.

### Cytometric bead array for cytokine analysis

The culture supernatants were harvested after the sorted CD4^+^ T cells were stimulated with PMA and ionomycin for 24 h. The cytokines in the culture supernatants and BALF were quantified using a Legendplex^TM^ kit (Cat # 740740; BioLegend) according to the manufacturer’s protocol.

### ELISA

To measure serum antibodies, we collected serum samples from mice via retro-orbital puncture on Day 14 after OVA challenge. A 96-well immunoplate was coated with 10 μg/mL OVA (Cat # A5503; MilliporeSigma) or goat anti-mouse IgG (Cat # 1010-01; Southern Biotech, AL, USA) and incubated overnight at 4 °C. After being blocked with 2% BSA in PBS, the plates were incubated with serially diluted standards or samples at a 1:20 dilution followed by horseradish peroxidase-conjugated detection antibodies (immunoglobulin (Ig) E, Cat # 1110-05; IgG, Cat # 1030-05; IgA, Cat # 1040-05; IgM, Cat # 1020-05; Southern Biotech) at a dilution of 1:1000. Subsequently, the plates were incubated with TMBE ELISA peroxidase substrate (Cat # TMBE-1000; Rockland Immunochemicals, Inc., PA, USA) for 15 min in the dark followed by 2 N H_2_SO_4_. The absorbance was then measured at 450 nm using a SPARK 10 M spectrophotometer (Tecan).

### Immunoblotting

Purified CD4^+^ T cells were lysed in radioimmunoprecipitation buffer (Cat # RC2002; Biosesang Inc., Gyeonggi-do, Republic of Korea) with a protease/phosphatase inhibitor (Cat # PPC1010; Millipore Sigma). The cell lysates were fractionated by SDS–PAGE and analyzed by immunoblotting using the following antibodies: p-AMPKα1 (Cat # ab23875) and AMPKα1 (Cat # Y365) from Abcam (Cambridge, UK); STAT6 (Cat # D3H4), SOCS1 (Cat # 3950T), SOCS2 (Cat # 2779T), SOCS3 (Cat # 52113T), SOCS5 (Cat # PA1-41253), GATA3 (Cat # 5852S), SIRT1 (Cat # 8469), p-AKT (Cat # S473, D9E), AKT (pan) (Cat # 1137), phospho-S6K (Thr398, Cat # 9205S), S6K (Cat # 49D7), p-SGK1 (Cat # D36D11), p-STAT3 (Cat # 9136S), STAT3 (Cat # 9139S), p-STAT1 (Cat # 9167S), STAT1 (Cat # 9172S), p-STAT4 (Cat # 4134S), STAT4 (Cat # 2653S), p-STAT5 (Cat # 4322S), STAT5 (Cat # 94205S), p-PKCθ (Cat # 9376S), PKCθ (Cat # 13643S), p-4EBP1 (Cat # 2855S), 4EBP1 (Cat # 9644S), and SGK1 (Cat # D27C11) from Cell Signaling Technology, Inc (MA, USA); and p-STAT6 (Cat # sc11762) from Santa Cruz Biotechnology, Inc. (CA, USA). β-actin (Cat # sc-47778) was purchased from Santa Cruz Biotechnology and used as the loading control. The blots were incubated with primary antibodies with continuous shaking overnight at 4 °C. The next day, the blots were washed with 1× TBST and incubated with secondary antibodies (1:1000 dilution) for 1 h at room temperature. The blot was again washed with 1× TBST, and the bands were visualized with West Pico Plus Chemiluminescent Substrate Kit (Cat # 34580; Thermo Fisher Scientific Inc.) and a LAS4000 luminescent analyzer (FUJIFILM Corporation, Tokyo, Japan). The immunoblots were quantified by densitometry using GelQuant software version 1.8.2, and the fold changes were calculated after normalization against the loading control β-actin.

### MitoTracker and tetramethylrhodamine methyl ester (TMRM) analyses

The total mitochondrial mass and mitochondrial membrane potential were analyzed by flow cytometry using MitoTracker^TM^ Red FM (Cat # M22425) and the MitoProbe^TM^ TMRM Assay kit (Cat # M20036) from Thermo Fisher Scientific Inc. For MitoTracker analysis, the cells were gently suspended in 50 µL of prewarmed (at 37 °C) MitoTracker probe and incubated at 37 °C for 3 min, followed by CD4 cell surface staining. Subsequently, the stained cells were analyzed by fluorescence-activated cell sorting (FACS) using an excitation/emission wavelength range of ~581/644 nm. Similarly, for the TMRM assay, cells were stained with 10 µM TMRM, as were the positive control (50 µM CCCP-treated) and negative control (DMSO-treated) cells. The cells were then incubated at 37 °C for 20 min in the dark and washed with 1× assay buffer. The prepared samples were analyzed using FACS with an excitation/emission wavelength range of ~488/561 nm.

### ATP measurement

For ATP measurement, the CellTiter-Glo Assay Kit (Cat # G7570; MilliporeSigma) was used according to the manufacturer’s protocol. First 100 µL of sorted CD4^+^ T cells (3 × 10^5^) were stimulated with anti-CD3 (2 µg/mL) and anti-CD28 antibodies (2 µg/mL) for 24 h at 37 °C in a 96-well opaque-walled flat bottom plate. After stimulation, 100 µL of Promega CellTiter-Glo reagent (Promega Corporation, WI, USA) was added to each sample well, incubated at 20–22 °C for 10 min, and mixed by pipetting. The luminescence was measured using a Tecan Infinite F200 fluorescence microplate reader (Tecan Group Ltd., Männedorf, Switzerland). The percentage of ATP change in *Prkaa1*^T-KO^ or *Sirt1*^T-KO^ CD4^+^ T cells was calculated relative to the luminescence value of WT CD4^+^ T cells.

### RT–PCR

Total RNA was isolated using the ReliPrep™ RNA Cell Miniprep system (Cat # Z6011; Promega Corporation), and cDNA was synthesized using the GoScript Reverse Transcription System (Cat # A500; Promega Corporation). The mRNA expression level of each gene was measured by real-time PCR using a QuantiTect SYBR Green PCR kit (QIAGEN GmbH Company, Hilden, Germany). The primer pairs used in this study are listed in Table 1. Relative gene expression was determined on the basis of C_t_ values. The ∆C_t_ was obtained after normalization against the reference gene (*Gapdh*), and fold changes were calculated relative to the WT control gene expression. The levels of *Gapdh* in samples remained unchanged between the different treatment conditions and cell types.

**Table 1:** Primers used for RT–PCR.

Gene	Forward	Reverse
*Hmgcr*	TGCTGCTTT GGCTGTATGTC	TGAGCGTGAACAAGAAGAACCAG
*Hmgcs1*	GTCCCTCCACAAATGACCAC	ATGACAGCCGACTCAGGTTC
*Idl1*	CACTGGCAGGAGTGATTGGA	TTGCTGGCATTGATTTCAGG
*Sqle*	TGGTGGAGGAATGACAGTCG	AAGCAAGCTTTTCGGAGCTG
*Got2*	GTTGAAATGGGACCTCCAGA	GGGCAGGTATTCTTTGTCCA
*Acox1*	AGCCTCTGCCAGGCATCAC	CATCAACATGTTCTCTCTAG
*Cpt2*	CAGTGCACAGAAGCCTCTCTTG	CTTCCCAATGCCGTTCTCAA
*Crat*	TGCTGCCAGAACCGTGGT	TCCAGGGATTGCTGAAGTGG
*Crot*	CGAACAGAGACTGTGCGATCTT	CATCTTTTGCTGACGTTCAAGG
*Acsl1*	ATCTGGTGGAACGAGGCAAG	TCCTTTGGGGTTGCCTGTAG
*Acsl3*	TGTCTTTCTCATGGATGCCGA	CAGCACGGATGTGTCTCCTT
*Bdh1*	AAGCACTGGAAGCAGACACAT	ACACTTAGGGCTTTTCCTGGG
*Hmgcl*	ACTACCCAGTCCTGACTCCAA	TAGAGCAGTTCGCGTTCTTCC
*Oxct2*	GTGGACGTGGGTACTTTCG	ACCATCTAGCAGAAGGAAGCTG
*Gk*	AGGGAACAACATCGTGGGAC	TCACATTGGCGGTCTTCATA
*Gpd1*	CTGAAGGACCTGATGCAGAC	GCTCAATGGACTTTCCAGTTC
*Lpl*	CAGAGTTTGACCGCCTTCC	AATTTGCTTTCGATGTCTGAGAA
*Aldh4a1*	CATAATCCAGTTTGTGCCAG	TCCACAGGTGTTTGAAGGTG
*Gls*	TTCGCCCTCGGAGATCCTAC	CCAAGCTAGGTAACAGACCCT
*Acat1*	GAAACCGGCTGTCAAAATCTGG	TGTGACCATTTCTGTATGTGTCC
*Acat2*	ACAAGACAGACCTCTTCCCTC	ATGGTTCGGAAATGTTCACC
*Acad1*	GCCTGGCCGGATTCATC	GGACATCACCCGTGTCTTCAT
*Mtor*	CTCAACATCGAGCATCGCATCATG	ACCAGAAAGGGCACCAGCCAATATAG
*Mlst8*	AGCGTGTGTTAAGTGCAGGT	AGTCCTGCTGCACTGTT
*Deptor*	AGCAGAGAGAGCTGGAACGC	CAGAGGCCTCCTTATGTTCA
*PRAS40*	TCAATACCAGCGACTTCCAGA	TGACCCTTGGAGCGTTTAGA
*Raptor*	AGATTGTGAAGGGGCTGACA	ACGCTTCTCCACCGAATACA
*Sin1*	TCGATTGTGACCTGCTCTGT	AAGCTTGTTCGCCTGTTCAG
*Rictor*	ACTTGTCCTCTGGCGCTTCA	AGCCTCACTTCATGCTTCCT

### Allergen-induced inflammation

We intranasally challenged WT, *Prkaa1*^T-KO^, and *Sirt1*^T-KO^ mice with 8 μL of *Aspergillus melleus* proteinase (2 mg/mL) (Millipore Sigma; Cat # P4032-5G) and 45 μL of ovalbumin (OVA; 1 mg/mL) (Millipore Sigma; Cat # A5503-10G). These nasal injections were repeated on Days 2, 4, 6, 10, and 12, and a booster dose was administered on Day 13 (proteinase 4 mg/mL, OVA 2 mg/mL). The mice were euthanized 24 h after the last challenge. Blood samples were collected on Day 14 to determine serum antibody levels. For the drug treatment experiments, AICAR (75 mg/kg/day) (Cat # 2840; Tocris Bioscience, Bristol, UK) and compound (Comp) C (5 mg/kg/day) (Cat # 171260; Millipore Sigma) were administered every other day beginning on Day 4 after the injection of proteinase-OVA.

### Bronchoalveolar lavage fluid (BALF)

We obtained BALF by washing the lungs with 500 μL of ice-cold PBS. The BALF was centrifuged, and the cell pellets were harvested and suspended in 500 μL of PBS. The supernatants were transferred to fresh tubes and frozen at −80 °C for cytokine analysis.

### Histology

At 24 h after the last proteinase/OVA challenge, the mice were euthanized, and the lung tissues were removed. The tissue was fixed in 4% paraformaldehyde (PFA), embedded in paraffin, and cut into 4-μm-thick sections. The sections were deparaffinized with xylene and stained with hematoxylin and eosin (H&E). The stained sections were analyzed microscopically to determine the extent of lung inflammation and goblet cell hyperplasia using a five-point grading system as described previously: 0, normal; 1, few cells; 2, ring of inflammatory cells 1-cell layer deep; 3, ring of inflammatory cells, 2–4 cells deep; and 4, ring of inflammatory cells, greater than 4 cells deep.

### Statistical analysis

The data are presented as the mean ± SEM of three independent experiments. The *P* values were calculated using Student’s *t* test or one-/two-way ANOVA with GraphPad Prism software (GraphPad Software, Inc., CA, USA), as specified in the figure legends. Differences were considered statistically significant at *P* ≤ 0.05.

## Results

### AMPK deficiency enhances Th2 differentiation and IL-4 signaling

**Fig. 1:**
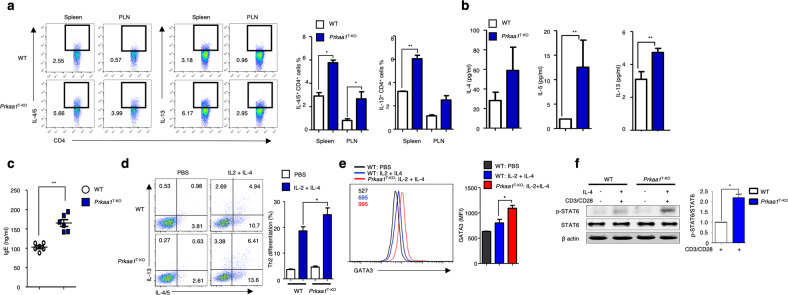
Prkaa1^T-KO^ mice exhibit a Th2 phenotype. **a** AMPK was selectively deleted in CD4^+^ T cells to generate *Prkaa1*^T-KO^ mice. Th2 responses were observed in 5-month-old *Prkaa1*^T-KO^ mice. The frequencies of IL-4/5- and IL-13-expressing CD4^+^ T cells in the spleen and peripheral lymph nodes (PLNs) of wild-type (WT) and *Prkaa1*^T-KO^ mice (*n* = 3). **b** IL-4, IL-5, and IL-13 secretion was assessed in ex vivo cultures of CD4^+^ T cells isolated from WT and *Prkaa1*^T-KO^ mice (*n* = 3). **c** Serum IgE levels in WT and *Prkaa1*^T-KO^ mice were measured by ELISA (*n* = 6). **d** In vitro Th2 differentiation of sorted naive WT and AMPK-deficient CD4^+^ T cells. **e** Representative histogram showing the helper T-cell transcription factor GATA3 in CD4^+^ T cells isolated from WT and AMPK-deficient mice and represented in the bar graph. The cells were cultured with or without IL-2 plus IL-4 stimulation. **f** Immunoblot analysis of STAT6 activation in WT and AMPK-deficient CD4^+^ T cells under resting and cytokine-stimulated conditions. The relative intensity and statistical analyses of TCR-stimulated phospho-STAT6 bands, which were normalized to the total STAT6 level, are shown in the bar graph. The data represent three independent experiments and are shown as the mean ± SEM. *P < 0.05, **P < 0.01.

To determine the role of AMPK in T cells, we generated T-cell-specific AMPK-deficient mice (hereafter referred to as *Prkaa1*^T-KO^ mice). Age-matched *Prkaa1*^+/+^CD4-Cre (WT) mice were used as controls. We further confirmed the specific deletion of AMPK in T cells from *Prkaa1*^T-KO^ mice using immunoblot analysis (Supplementary Fig. 1a). Subsequent investigations revealed that *Prkaa1*^T-KO^ mice were born at the expected Mendelian ratios and appeared grossly normal at a young age. The spleen and lymph nodes were comparable in size to those of WT and *Prkaa1*^T-KO^ mice (data not shown). Furthermore, *Prkaa1*^T-KO^ mice exhibited no significant changes in the frequencies of T_eff_, T_reg_, Th1, and Th17 CD4^+^ T cells or in total CD4^+^ and CD8^+^ cell counts in the spleen, MLNs, and peripheral lymph nodes (PLNs). (Supplementary Fig. 1b–f). However, the percentages of IL-4^+^, IL-5^+^, and IL-13^+^ CD4^+^ T cells were increased in the spleen and PLNs of *Prkaa1*^T-KO^ mice compared with WT mice (Fig. 1a). Moreover, CD4^+^ T cells from *Prkaa1*^T-KO^ mice secreted increased amounts of signature Th2 cytokines (IL-4, IL-5, and IL-13) during ex vivo culture (Fig. 1b). In addition, basal serum IgE levels were increased in *Prkaa1*^T-KO^ mice (Fig. 1c), whereas IgG, IgM, and IgA levels were comparable to those in WT mice (Supplementary Fig. 1g). When naive CD4^+^ T cells were subjected to Th1- or Th17-skewing conditions, the frequencies of IFN-γ^+^ Th1 and IL-17A^+^ Th17 cells were comparable between WT and AMPK-deficient CD4^+^ T cells (Supplementary Fig. 1h, i). However, the frequencies of IL-4^+^, IL-5^+^, and IL-13^+^ Th2 cells were significantly higher in AMPK-deficient CD4^+^ T cells than in WT CD4^+^ T cells in response to Th2-skewing conditions (Fig. 1d). Surprisingly, AMPK activity was decreased in Th2 cells and was unchanged in Th1 and Th17 cells (Supplementary Fig. 1j). In addition to Th2-biased differentiation, our results showed that the expression of the Th2 transcription factor GATA3 was increased in AMPK-deficient CD4^+^ T cells compared with WT CD4^+^ T cells under Th2-skewing conditions (Fig. 1e), whereas the levels of T-bet and RORγt were comparable under the Th1- and Th17-skewing conditions (Supplementary Fig. 2a, b). Furthermore, IL-4 has been reported to initiate the Th2 differentiation signal that induces the phosphorylation and activation of STAT6, which in turn enhances GATA3 expression^15^. Consequently, we analyzed the activity of the STAT6 protein, which has been reported to be phosphorylated through IL-4R signaling^2,3^. As expected from the Th2-biased characteristics of AMPK-deficient CD4^+^ T cells, we found elevated phosphorylation of STAT6 in response to IL-4 stimulation compared with that in WT CD4^+^ T cells (Fig. 1f). In contrast, the phosphorylation of STAT3 in response to IL-6 stimulation, STAT1 and STAT4 in response to IL-12 stimulation, and STAT5 in response to IL-2 cytokine stimulation was unchanged (Supplementary Fig. 2c–e). Taken together, our results indicate that AMPK regulates the development of Th2 cells but not the differentiation of Th1/Th17 CD4^+^ T cells.

### AMPK deficiency enhances mTORC2 signaling

**Fig. 2:**
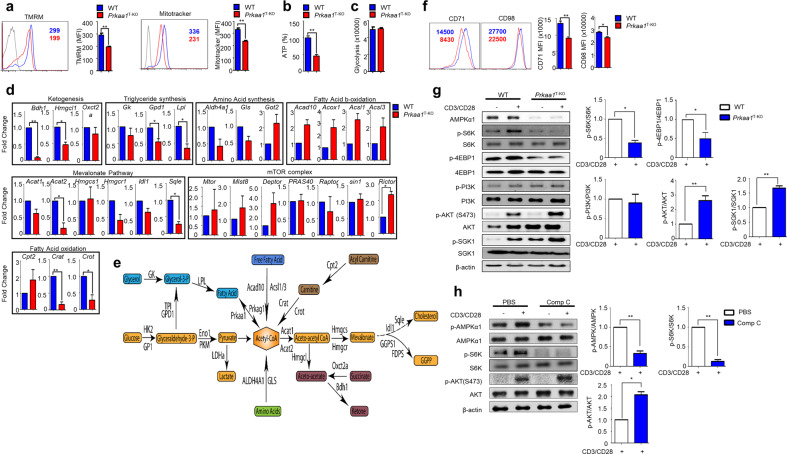
AMPK deficiency alters metabolic signatures in CD4^+^ T cells. **a** Histograms of the tetramethylrhodamine methyl ester (TMRM) and MitoTracker (*n* = 3) assay results. **b, c** ATP percentage (**b**) and glycolysis (**c**) in wild-type (WT) and AMPK-deficient CD4^+^ T cells (*n* = 3). **d** The metabolic pathways of related genes were assessed by RT–PCR. **e** Schematic representation of pathways and regulatory genes. **f** The activation markers CD71 and CD98 in WT and AMPK-deficient CD4^+^ T cells. **g** Immunoblot analysis of AMPKα1, p-S6K, S6K, p-4EBP1, 4EBP1, p-PI3K, PI3K, p-AKT(S473), AKT, p-SGK1, SGK1, and the loading control β-actin in WT and AMPK-deficient CD4^+^ T cells under resting and stimulated conditions. The relative intensities and statistical analyses of TCR-stimulated p-S6K bands normalized to S6K, p-PI3K bands normalized to PI3K, p-AKT bands normalized to AKT, and p-SGK1 bands normalized to SGK1 are shown in the bar graphs. **h** Immunoblot analysis of p-AMPKα1, AMPKα1, p-S6K, S6K, p-AKT, AKT, and the loading control β-actin in WT CD4^+^ T cells treated with/without compound (Comp) C under resting and TCR-stimulated conditions. The relative intensities and statistical analyses of TCR-stimulated phospho-protein bands normalized to the total protein bands are shown in the bar graphs. The data represent three independent experiments, with the mean ± SEM. *P < 0.05, **P < 0.01.

AMPK is involved in energy utilization, lipid and protein metabolism, and mitochondrial activity in many different cell types^16^. Because AMPK is linked to T-cell energy metabolism, we found a marked decrease in mitochondrial membrane potential and basal ATP levels in AMPK-deficient CD4^+^ T cells compared with WT cells (Fig. 2a, b). In contrast, we observed comparable glycolytic activity in WT and AMPK-deficient CD4^+^ T cells (Fig. 2c). Previous studies on T cells have also shown reduced oxygen consumption rates and unaltered extracellular acidification rates in AMPK-deficient T cells^8,17^. These results suggest that AMPK-deficient CD4^+^ T cells are unable to sustain cellular energy and have defects in mitochondrial function, resulting in reduced ATP generation. Previous studies have also suggested that AMPK-mediated metabolism of proteins, lipids, and carbohydrates plays an important role in coordinating T-cell proliferation and function^18^. However, the mechanism by which AMPK regulates each metabolic pathway remains unclear. To determine the metabolic pathway involved in Th2 differentiation, we first examined the expression of representative genes involved in major metabolic pathways. The results revealed that the expression levels of multiple genes related to ketogenesis, fatty acid oxidation, the mevalonate pathway, and triglyceride synthesis were decreased, whereas those related to fatty acid β-oxidation were slightly upregulated in AMPK-deficient CD4^+^ T cells compared with WT CD4^+^ T cells (Fig. 2d, e). In contrast, mTORC2 activity was increased in AMPK-deficient CD4^+^ T cells (Fig. 2d). mTORC2 is an important contributor to the cytoskeleton, cell survival, and cellular metabolism^19^. An earlier study reported that the LKB1–AMPK axis negatively regulates mTORC1 signaling^20^. However, mTORC1 signaling was not enhanced by AMPK deletion in CD4^+^ T cells (Fig. 2d, f). Furthermore, the expression levels of CD71 and CD98, which are mTORC1-associated transporters, were reduced in AMPK-deficient CD4^+^ T cells (Fig. 2f). We also confirmed that the phosphorylation of S6K and 4E-BP1 (Fig. 2g), which are downstream targets of mTORC1, was significantly decreased in AMPK-deficient T cells. Thus, our results indicate that AMPK did not downregulate mTORC1 signaling. Instead, AMPK deletion enhanced the phosphorylation of mTORC2 target molecules, including AKT (S473) and SGK1, in CD4^+^ T cells (Fig. 2g). These results were further validated by treating WT CD4^+^ T cells with Comp C (a pharmacological inhibitor of AMPK) and TCR stimulation. Our findings revealed that a weaker AMPK response after Comp C treatment resulted in increased phosphorylation of AKT (S473) (Fig. 2h). Overall, these results suggest that AMPK is a negative regulator of signaling associated with mTORC2 but not mTORC1.

### AMPK controls allergen-induced allergic inflammation

**Fig. 3:**
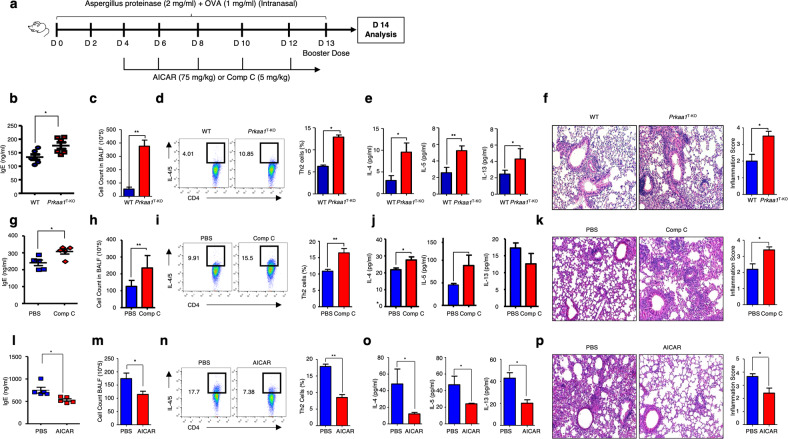
AMPK controls allergen-induced allergic inflammation. **a** Schematic diagram showing the experimental design for allergen-induced inflammation in vivo. Mouse background and drug treatment may differ according to the experiment. Allergic inflammation was induced in wild-type (WT) mice by intranasal challenge with proteinase-OVA. Analysis was performed on Day 14. **b**‒**f** WT (wild-type) and *Prkaa1*^T-KO^ mice were intranasally challenged with *Aspergillus melleus* proteinase/OVA. The analysis was performed on Day 14. OVA-specific serum IgE levels (**b**). BALF cell counts (**c**). Th2 cell percentages in mediastinal lymph nodes (**d**). Th2 cytokine levels in BALF (**e**). Hematoxylin and eosin-stained lungs (**f**) (*n* = 6). **g**‒**p** C57BL/6 mice treated with or without compound (Comp) C (**g**–**k**) and Balb/c mice treated with or without AICAR (**l**–**p**). OVA-specific serum IgE (**g**, **l**), BALF cell counts (**h**, **m**), Th2 cell percentages in mediastinal lymph nodes (**i**, **n**), Th2 cytokine levels in BALF (**j**, **o**), and histological analysis of the lungs (**k**, **p**) (*n* = 5). The data are representative of three independent experiments and are shown as the mean ± SEM. *P < 0.05, **P < 0.01.

Since Th2 and mTORC2 signaling were elevated in AMPK-deficient CD4^+^ T cells, we use *A*. proteinase/OVA-induced inflammation in *Prkaa1*^T-KO^ mice as a Th2-mediated disease model (Fig. 3a). This experimental model induced allergen-specific IgE and Th2 cell differentiation, which are representative hallmarks of allergic inflammation^21^. Our results showed that on Day 14 after allergen-induced inflammation, *Prkaa1*^T-KO^ mice showed increased inflammation, including serum IgE antibodies (Fig. 3b), overall cellularity in BALF (Fig. 3c), and Th2 frequency in mediastinal lymph nodes (MdLNs) (Fig. 3d). Consistent with the increase in Th2 cells, pulmonary IL-4, IL-5, and IL-13 levels were also higher in the BALF from *Prkaa1*^T-KO^ mice than in the BALF from control mice (Fig. 3e). Histological analysis revealed thicker basement membranes with increased numbers of immune cells in the lungs of *Prkaa1*^T-KO^ mice (Fig. 3f) than in the lungs of control mice. Thus, these results suggest that AMPK regulates Th2 cell development and related cytokine production when stimulated with allergic inflammation.

To confirm the role of AMPK, mice with allergen-induced inflammation were treated with pharmacological inhibitors and activators of AMPK. Subsequent analyses revealed that global inhibition of AMPK by Comp C enhanced the pathogenesis of allergic inflammation (Fig. 3g–j) and increased lung inflammation (Fig. 3k). Thus, we concluded that genetic deletion or pharmacological inhibition of AMPK augmented the Th2 response and Th2 cytokine production in vivo in the context of allergic inflammation. Next, we treated the mice with AICAR, an AMPK activator, to determine whether AMPK activation attenuates allergic inflammation. The results revealed that AICAR reduced IL-4 levels in BALF without affecting the inflammatory phenotypes (Supplementary Fig. 3a–d). As BALB/c mice exhibited a more Th2-dominant environment than other mice^22^, we treated allergen-induced inflammatory BALB/c mice with AICAR. As expected, BALB/c mice exhibited stronger basal allergic phenotypes than C57BL/6 mice, and treatment with AICAR reduced all the tested allergic inflammatory phenotypes (Fig. 3l–p). Furthermore, AICAR-treated BALB/c mice also showed reduced cellular infiltration in the basement membranes of the lungs compared with mice in the control group (Fig. 3p). Overall, these results indicate that AMPK negatively regulates Th2 responses during allergic inflammation in mice.

### SIRT1 acts upstream of AMPK to regulate the mTORC2 pathway

Next, we investigated the molecular pathways involved in AMPK-dependent Th2 regulation. Liver kinase B1 (LKB1) is an upstream regulator of the AMPK signaling cascade in most mammalian cells^23^. However, recent studies on regulatory T cells (T_regs_) have shown that AMPK acts independently of LKB1^13^. Moreover, LKB1 could negatively regulate T-cell effector functions through mTORC1^11^. These findings were further corroborated by a report showing that LKB1 negatively regulates mTORC1 but not mTORC2 through LKB1-PTEN binding in CD4^+^ T cells^24^. The NAD-dependent deacetylase SIRT1 is associated with AMPK^25^. SIRT1 has been shown to deacetylate STAT3 to suppress Th9 differentiation in human CD4^+^ T cells^26,27^. However, its role in Th2 cell differentiation remains unknown. To determine whether SIRT1 mediates AMPK-dependent mTORC2 activation in CD4^+^ T cells, we generated CD4^+^ T-cell-specific *Sirt1* gene-knockout (*Sirt1*^T-KO^) mice and used immunoblot analysis to assess SIRT1 protein expression in purified CD4^+^ T cells from WT and *Sirt1*^T-KO^ mice with and without TCR stimulation (Supplementary Fig. 4a). Further analysis revealed that similar to *Prkaa1*^T-KO^ mice, *Sirt1*^T-KO^ mice at a steady state had levels of CD4^+^, CD8^+^, T_eff_, T_reg_, Th1, Th17, and total CD4^+^ and CD8^+^ cells that were comparable to those in WT mice (Supplementary Fig. 4b–f). Furthermore, the cytokine profiles of *Prkaa1*^T-KO^ and *Sirt1*^T-KO^ mice showed skewed Th2 characteristics (Supplementary Fig. 5). However, mitochondrial metabolism and ATP generation in *Sirt1*-deficient CD4^+^ cells were comparable to those in WT CD4^+^ cells (Supplementary Fig. 6). These results suggest that AMPK and SIRT1 act independently of mitochondrial metabolism in murine CD4^+^ T cells.

**Fig. 4:**
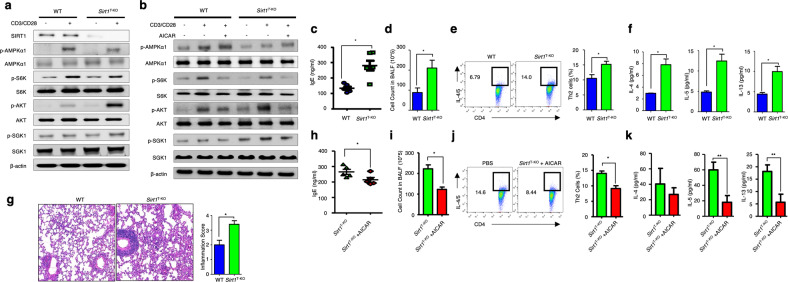
The loss of SIRT1 increases allergic inflammation. **a** Immunoblot analysis of SIRT1, p-AMPKα1, AMPKα1, p-S6K, S6K, p-AKT, AKT, p-SGK1, SGK1, and the loading control β-actin in *Sirt1-*deficient CD4^+^ T cells under resting and stimulated conditions. The blots were quantified by densitometry and are shown in the bar graph in Supplementary Fig. 7a. **b** Immunoblot analysis of p-AMPKα1, AMPKα1, p-S6K, S6K, p-AKT, AKT, p-SGK1, SGK1, and the loading control β-actin in *Sirt1*-deficient CD4^+^ T cells with or without in vitro AICAR treatment under resting and TCR-stimulated conditions. Blots were quantified and are shown in the bar graph in Supplementary Fig. 7b. **c**–**g** Allergic inflammation was induced in WT and *Sirt1*^T-KO^ mice. **h**–**k** Allergic inflammation was induced in *Sirt1*^T-KO^ mice with/without intraperitoneal AICAR treatment. OVA-specific serum IgE levels (**c**, **h**), BALF cell counts (**d**, **i**), Th2 cell percentages in mediastinal lymph nodes (**e**, **j**), Th2 cytokine levels in BALF (**f**, **k**), and histological analysis of the lungs (**g**). The data analysis for all groups was performed on Day 14 (*n* = 5). The data shown are from three independent experiments and are presented as the mean ± SEM. *P < 0.05, **P < 0.01.

**Fig. 5:**
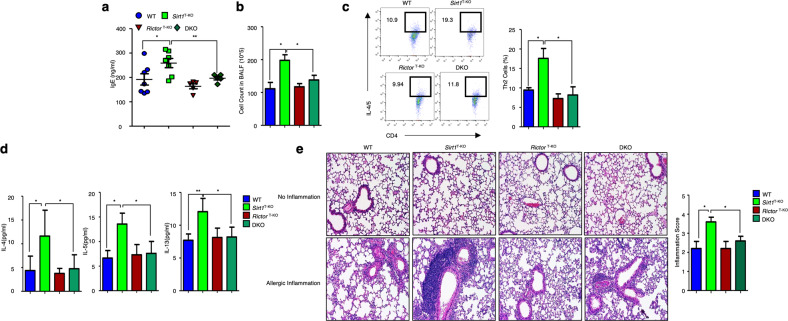
The SIRT1–AMPK axis is an upstream regulator of mTORC2 signaling. **a**‒**e** Allergic inflammation was induced by intranasal injection of proteinase-OVA in wild-type (WT), *Sirt1*^T-KO^, *Rictor*^T-KO^, and DKO mice. Analysis was performed on Day 14. OVA-specific serum IgE levels (**a**). BALF cell counts (**b**). Th2 cell percentages in mediastinal lymph nodes (**c**). Th2 cytokine levels in BALF (**d**). Histologic analysis of the lungs (**e**). The data represent three independent experiments and are shown as the mean ± SEM. *P < 0.05, **P < 0.01.

In addition, activated *Sirt1*-deficient CD4^+^ T cells showed reduced AMPK phosphorylation compared with that in the WT CD4^+^ T cells (Fig. 4a and Supplementary Fig. 7a). Similar to AMPK-deficient CD4^+^ T cells, *Sirt1*-deficient CD4^+^ T cells also exhibited increased mTORC2 activation (Fig. 4a and Supplementary Fig. 7a). Furthermore, stimulating *Sirt1*-deficient CD4^+^ T cells with AICAR, a potent activator of AMPK in CD4^+^ T cells, resulted in the suppression of mTORC2 activity (Fig. 4b and Supplementary Fig. 7b). To investigate whether increased mTORC2 activity induced the Th2-biased phenotype, we induced allergic inflammation in WT and *Sirt1*^T-KO^ mice and confirmed the increase in allergic phenotypes in *Sirt1*^T-KO^ mice compared with WT mice (Fig. 4c–f). In addition, the lungs of allergic *Sirt1*^T-KO^ mice showed more immune cell infiltration and thicker basement membranes than the lungs of WT mice (Fig. 4g). Notably, AICAR-induced activation of AMPK in *Sirt1*^T-KO^ mice attenuated allergic inflammation (Fig. 4h–k). Overall, these results indicate that SIRT1 acts upstream of AMPK and controls Th2-mediated allergic inflammation by regulating the mTORC2 pathway. To verify that the SIRT1–AMPK axis controls allergic inflammation by suppressing mTORC2 activation, we tried to generate knockout mice that were deficient in both AMPK and Rictor in CD4-expressing cells, as Rictor is an important protein involved in the regulation of mTORC2 activity^28^. However, the double-knockout mouse generation was unsuccessful because *Prkaa1* and *Rictor*, which encode AMPK and Rictor, respectively, are located on chromosome 15 between two loci that are approximately 1 cm apart, which gives an approximately 1% chance of meiotic recombination between the homologous chromosomes^29^. Due to this low chance of recombination, we were unable to generate AMPK and *Ricto*r DKO mice. Instead, we generated CD4^+^ T-cell-specific *Sirt1* and *Rictor* DKO mice to investigate the role of these genes in AMPK-regulated allergic inflammation. As expected, DKO of *Sirt1* and *Rictor* reversed the increased Th2 response in the *Sirt1*^T-KO^ mice by eliminating mTORC2 activation (Fig. 5a–e). Taken together, these results suggest that the increase in allergic inflammation in *Prkaa1*^T-KO^ and *Sirt1*^T-KO^ mice is attributed to the suppression of mTORC2 signaling.

### AMPK suppresses IL-4 signaling via SOCS5

**Fig. 6:**
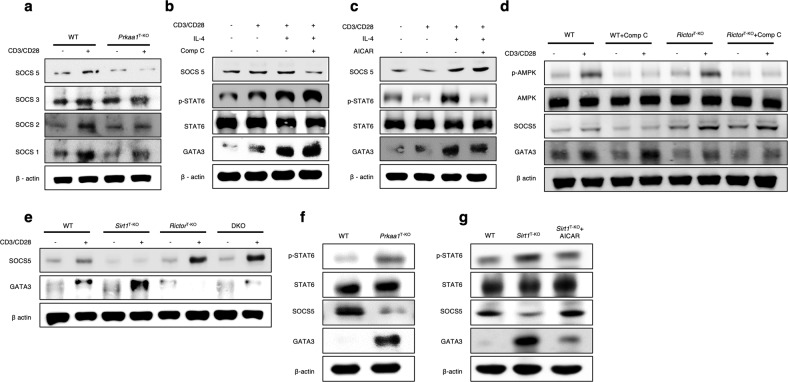
SOCS5 suppresses GATA3 expression. **a** Immunoblot analysis of the expression levels of SOCS1, SOCS2, SOCS3, and SOCS5 in wild-type (WT) and AMPK-deficient CD4^+^ T cells under resting and TCR-stimulated conditions. β-actin was used as a loading control. The quantified blots are shown as bar graphs in Supplementary Fig. 8a. **b**, **c** Immunoblot analysis of SOCS5, p-STAT6, STAT6, GATA3, and the loading control β-actin with or without TCR stimulation, and IL-4 cytokine, and compound (Comp) C (**b**) or AICAR treatment (**c**). The quantified blots are presented in the bar graphs in Supplementary Fig. 8b, c. **d** Immunoblot analysis of phosphorylated and total AMPK, SOCS5, GATA3, and the loading control β-actin in resting and TCR-stimulated WT and *Rictor-*deficient CD4^+^ T cells with or without Comp C treatment. The quantified blots are presented in the bar graphs in Supplementary Fig. 8d. **e** Immunoblot analysis of SOCS5, GATA3, and the loading control β-actin in resting and TCR-stimulated WT, *Sirt1*-deficient, *Rictor*-deficient, and DKO CD4^+^ T cells. The quantified blots are represented as bar graphs in Supplementary Fig. 8e. **f**, **g** Allergic inflammation was induced in WT, *Prkaa1*^T-KO^ (**f**), WT, and *Sirt1*^T-KO^ mice with or without AICAR treatment (**g**). Immunoblot analysis of phosphorylated and total STAT6, SOCS5, GATA3, and the loading control β-actin in CD4^+^ T cells that were stimulated overnight with OVA. The quantified blots are represented in the bar graphs in Supplementary Fig. 8f, g. The data are representative of three independent experiments.

Because mTORC2 signaling activation and STAT6 phosphorylation positively regulate Th2 cell differentiation^3,6^, we investigated how mTORC2 activation increases STAT6-mediated IL-4 signaling under AMPK-deficient conditions. SOCS family members have been reported to regulate IL-4 signaling^5^. Thus, we examined the levels of SOCS proteins in AMPK-deficient CD4^+^ T cells. The results revealed that the expression levels of SOCS1, SOCS2, and SOCS5 were reduced in activated AMPK-deficient CD4^+^ T cells compared with WT cells, while the levels of SOCS3 remained unaffected (Fig. 6a and Supplementary Fig. 8a). In addition, SOCS5 protein expression was decreased in Comp C-treated CD4^+^ T cells but increased in AICAR-treated cells (Fig. 6b, c and Supplementary Fig. 8b, c). SOCS5 negatively regulates IL-4-mediated STAT6 activation^5^, which was corroborated by the negative correlation between the SOCS5 expression level and STAT6 activation in the activated CD4^+^ T cells in the present study (Fig. 6b, c and Supplementary Fig. 8b, c). Similarly, the expression of the Th2 transcription factor GATA3 was negatively correlated with SOCS5 expression (Fig. 6b, c and Supplementary Fig. 8b, c). These data indicate that SIRT1–AMPK signaling regulates STAT6-mediated Th2 differentiation by activating SOCS5. We next determined that *Rictor*-deficient CD4^+^ T cells exhibit elevated SOCS5 levels and decreased GATA3 expression (Fig. 6d and Supplementary Fig. 8d). In addition, Comp C failed to increase GATA3 expression in *Rictor*-deficient CD4^+^ T cells (Fig. 6d and Supplementary Fig. 8d). Furthermore, a decrease in SOCS5 and an increase in GATA3 were observed in *Sirt1*-deficient CD4^+^ T cells but not in DKO CD4^+^ T cells (Fig. 6e and Supplementary Fig. 8e). Taken together, these data suggest that AMPK supports SOCS5 activation by negatively regulating mTORC2, which ultimately regulates IL-4-dependent STAT6 activation during Th2 differentiation.

To confirm that the Sirt1–AMPK axis regulates mTORC2 via SOCS5 in vivo, allergic inflammation was induced in *Prkaa1*^T-KO^ and *Sirt1*^T-KO^ mice. Similar to the in vitro results, SOCS5 expression was decreased while GATA3 expression was increased in AMPK- and *Sirt1*-deficient CD4^+^ T cells from allergic inflammation-induced *Prkaa1*^T-KO^ and *Sirt1*^T-KO^ mice, respectively (Fig. 6f, g and Supplementary Fig. 8f, g), and AICAR treatment rescued the expression of SOCS5 in *Sirt1*-deficient cells (Fig. 6g and Supplementary Fig. 8g). These findings suggest that the SIRT1–AMPK axis supports SOCS5 activation by negatively regulating mTORC2 activation under allergic inflammatory conditions.

## Discussion

Allergen-induced inflammation generates a Th2 cell response, and increased IL-4 and IL-13 levels drive the production of IgE by promoting B-cell’s immunoglobulin class switching^30^. Multiple pathways coordinate during immune cell differentiation, and accumulating evidence suggests metabolic involvement in this process. AMPK is a master metabolic regulator in mammalian cells, including immune cells^31^. As cellular metabolism plays a vital role in the proliferation and effector differentiation of CD4^+^ T cells^32^, we investigated the role of AMPK in CD4^+^ T cells. Although metabolic reprogramming is vital for effector T-cell subset differentiation and function^33^, *Prkaa1*^fl/fl^CD4-Cre (AMPK^T-KO^) mice showed an exclusively Th2-biased phenotype. Furthermore, the unaltered Th1 and Th17-cell populations in AMPK^T-KO^ mice suggest an alternative mode of AMPK signaling beyond metabolism, which regulates Th2 differentiation. Interestingly, a study on CD4^+^ T-cell subsets revealed increased AMPK activity in Tregs, whereas decreased AMPK activity were evident in Th2 cells, which is consistent with our observations^34^. An earlier study reported that the transcription factor c-Maf (large Maf protein) was exclusively elevated in Th2 cells^35,36^. Furthermore, a study on pancreatic β-cells showed that c-Maf expression levels could be increased by inhibiting AMPK^36^. In addition, the differentiation of Th1 and Th17 cells has been reported to be regulated by the LKB1/PTEN axis in an AMPK-independent manner^24^. AMPK is an energy sensor that negatively regulates the mTORC1 pathway^37^. It also controls mTORC2 expression in nonimmune cells. For example, AMPK-dependent phosphorylation of mTOR has been reported to promote mTORC2 signaling in mouse embryonic fibroblasts^38^ and cancer cell lines^39^. In contrast, AMPK has also been shown to inhibit mTORC2 signaling by phosphorylating TSC2, which in turn downregulates the phosphorylation of mTOR in myeloma cells^40^ and non-small cell lung cancer cells^41^.

The oral diabetes drug metformin, which activates AMPK, has been reported to be associated with decreased allergic airway inflammation and chronic obstructive pulmonary disease (COPD) in humans^42,43^. Furthermore, metformin attenuates the exacerbation of OVA-induced allergic inflammation in obese mice by reducing eosinophilic recruitment^44^, which can be further differentiated based on the expression of cytokines and chemokines secreted by Th2 cells^45^. In a previous study, AICAR-mediated activation of AMPK was shown to exert an inhibitory effect on the expression of Th2 cytokines (IL-5 and IL-13) and transcription factors (GATA3) by reducing HuR function^46^.

A previous study on hepatocytes reported that metformin-induced AMPK negatively regulates the mTORC1 pathway^47^. Furthermore, the activation of mTORC1 and inflammatory cytokine production was observed in AMPK-deficient CD8^+^ T cells but not in CD4^+^ T cells^8,11^, suggesting diverse roles of AMPK in T-cell subtypes. An earlier study reported that AMPK deficiency did not affect T-cell fate decisions and activation; however, AMPK-deficient T cells showed increased phosphorylation of TSC2 and Akt signaling^17^. Recently, the LKB1–AMPK signaling pathway was shown to act independently in CD4^+^ and T_reg_ cells^13,24^. Unlike the LKB1^T-KO^ mouse phenotype^24^, *Prkaa1*^T-KO^ mice did not show hyperactivated or inflammatory phenotypes in the steady state. In addition, mTOR activity, as well as CD71 and CD98 expression, were downregulated in TCR-stimulated AMPK-deficient CD4^+^ T cells^24^. In the present study, mTORC1 signaling was downregulated in AMPK-deficient CD4^+^ T cells, and mTORC2 signaling was upregulated. These findings contradict the findings of a previous study suggesting that mTORC2 phosphorylates AKT, which subsequently activates the mTORC1 pathway^48^. However, it has also been suggested that mTORC1 and mTORC2 regulate each other via a negative feedback mechanism, depending on the metabolic status of the cell^49^, which might be explain why AMPK-deficient CD4^+^ T cells showed increased Th2 differentiation but not Th1 or Th17 differentiation. These findings correlate with a recent report demonstrating that mTORC1 regulates Th1/Th17 differentiation^24^. Further studies are needed to determine the relationship between the kinases LKB1 and AMPK in CD4^+^ T-cell biology.

In contrast to previous studies, we found slightly upregulated expression of β-oxidation-related genes in AMPK-deficient CD4^+^ T cells. β-Oxidation leads to the production of acetyl-CoA, which has been reported to be enhanced in cancer cells, along with an increase in the mTORC2 pathway^50^. Furthermore, Um et al. reported that mice that were deficient in S6K1 showed enhanced β-oxidation^51^. In our mouse model, mTORC2 activity was increased, and the expression of S6K1 was decreased in AMPK-deficient CD4^+^ T cells, which may cause the slight increase in fatty acid β-oxidation. However, these subtle differences warrant further clarification.

Several earlier studies have shown a critical role of mTORC2 in Th2 cell differentiation^6,52,53^; however, the upstream regulator of mTORC2 signaling in CD4^+^ T cells has not yet been determined. In the present study, we showed that the SIRT1–AMPK axis was a negative regulator that acts upstream of mTORC2, and the loss of these molecules in CD4^+^ T cells exacerbated allergic inflammation. A previous study reported that SIRT1-deficient CD4^+^ T cells resulted in worsened OVA-induced allergic airway inflammation^27^. It is therefore possible that lower SIRT1 expression levels are associated with more severe outcomes in allergic patients^54^. In contrast, SIRT1 increases the expression of mTORC2 components in hepatocytes^55^. These findings show that CD4^+^ T cells use metabolic pathways in distinct ways. Other studies have suggested that SIRT1 overexpression activates AMPK in embryonic kidney cells and hepatocytes through the deacetylation of LKB1^56–58^. In contrast, LKB1 is dispensable for AMPK-mediated mTORC2 activation (data not shown). To confirm the regulation of mTORC2 by AMPK (in full awareness of the negligible chances of generating AMPK and *Rictor* DKO, as mentioned in the results), we attempted to generate CD4-specific *Sirt1* and *Rictor* DKO mice, since SIRT1 acts as an upstream kinase of AMPK^59^. Consequently, we observed that the DKO mice successfully blunted the excess Th2 response during allergen-induced inflammation. Taken together, these results provide important evidence about the role of SIRT1–AMPK signaling, through which Th2 responses are regulated.

A previous study on *Lck*^Cre^*Rictor*^fl/fl^ mice showed the regulation of Th2 via PKC-θ activity, and the transduction of a constitutively active PKC-θ mutant restored the Th2 cell defect in *Rictor*-deficient CD4^+^ T cells independent of STAT6 activity^60^. However, in our study, we observed enhanced IL-4R/STAT6 signaling during Th2 cell differentiation. Furthermore, PKC-θ-deficient T cells exhibit normal STAT6 activity and Th2 differentiation^61^. Thus, in our study, we focused on investigating the role of SOCS5, which regulates the differentiation of Th2 cells via STAT6 activity^6^. Although these contrasting observations remain unclear and require further investigation, we hypothesize that these subtle differences may be due to the different knockout mouse models used in these studies.

Finally, we investigated the mechanism by which mTORC2 augments the differentiation of Th2 cells. IL-4 has been shown to trigger IL-4R/STAT6 signaling, resulting in Th2 cell differentiation via the transcription factor GATA3 in an autocrine manner^2,3^. Moreover, IL-4 signaling is negatively regulated by SOCS proteins in CD4^+^ T cells^5^ and other cell types, such as monocytes and macrophages^4^, as well as HEK293 cells^62^. In this study, we found that the expression of SOCS1, SOCS2, and SOCS5 was reduced in AMPK-deficient CD4^+^ T cells, whereas the expression of mTORC2 was upregulated. In addition, mTORC2 inhibits SOCS5 expression via Kruppel-like factor 2 (KLF2) after AKT activation in CD4^+^ T cells^63^. These findings clearly suggest that mTORC2 suppresses the expression of SOCS proteins and facilitates the induction of IL-4 signaling.

**Fig. 7:**
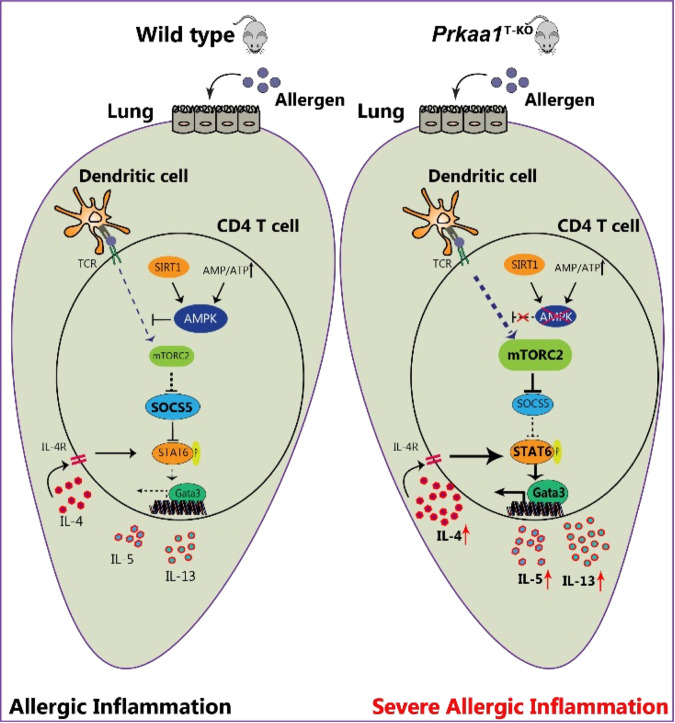
The role of AMPK in suppressing Th2-induced allergic inflammation in CD4^+^ T cells. Schematic diagram showing that AMPK deficiency in CD4^+^ T cells is unable to suppress mTORC2 activity, which inhibits SOCS5 expression. This reduction in SOCS5 protein levels leads to a failure to suppress the IL-4-STAT6–GATA3 signaling axis, resulting in severe allergic inflammation.

In summary, our study revealed a crucial role of AMPK in the differentiation of Th2 cells and allergic inflammation. We showed that metabolic activation of AMPK, such as by AICAR treatment, could suppress the mTORC2 response in CD4 T^+^ cells. We also found that the loss of AMPK signaling decreased SOCS5 expression, which in turn resulted in its failure to suppress the IL-4–STAT6–GATA3 signaling axis, leading to increased Th2 cell differentiation (Fig. 7).

## Supplementary information

Supplementary Figure 1-8

## Data Availability

The data supporting the findings of this study are available from the corresponding author upon reasonable request.
