# Supplementary material for: AMPK suppresses Th2 cell responses by repressing mTORC2
Source: Exp Mol Med. 2022 Aug 23;54(8):1214–24. doi: 10.1038/s12276-022-00832-x (PMC9440126; doi:10.1038/s12276-022-00832-x)
Supplement: Supplementary file 1 — Supplementary Figure 1-8 [file 12276_2022_832_MOESM1_ESM.pdf]

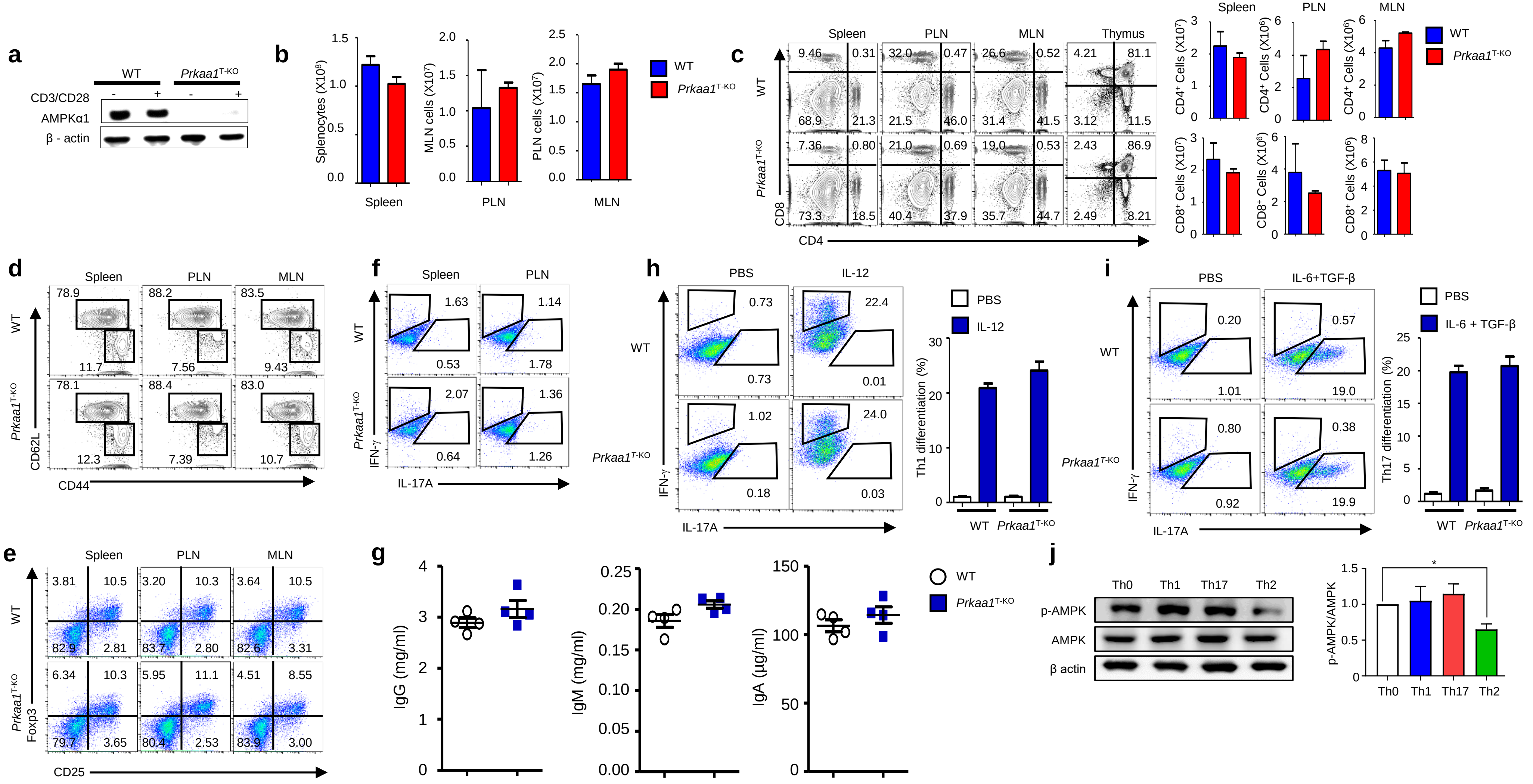

Supplementary Fig 1. *Prkaa1*$^{T\text{-}KO}$ mice have normal T cell phenotype. **a** Immunoblot analysis of AMPKα1 protein expression and β-actin as loading control in purified CD4$^+$ T cells from wild-type (WT) and *Prkaa1*$^{T\text{-}KO}$ mice with/without CD3/CD28 stimulation. **b–f** Cells isolated from spleen, peripheral and mesenteric lymph nodes (PLN and MLN), and thymus of 8-week-old WT and *Prkaa1*$^{T\text{-}KO}$ mice. Cell counts were done by hemocytometer and sub-populations of indicated tissues were analyzed by FACS. Spleen, MLN, and PLN total cell counts (**b**). CD4$^+$ and CD8$^+$ T cells frequencies and counts for indicated lymphoid tissues (**c**). Naïve (CD62L^{high}CD44^{low}) and effector memory (CD62L^{low}CD44^{hi}) CD4$^+$ T cells (**d**). CD25$^+$Foxp3$^+$ T$_{reg}$ cells (**e**). IFN-γ$^+$ (Th1) and IL-17A$^+$ (Th17) CD4$^+$ T cells (**f**). **g** Serum IgG, IgM and IgA levels of WT and *Prkaa1*$^{T\text{-}KO}$ mice measured by ELISA. **h-i** In vitro differentiation of Th1 and Th17 cells. **j** Immunoblot analysis for p-AMPK and AMPK were done and represented in bar graph. Results represent three independent experiments. Data are mean ± SEM.

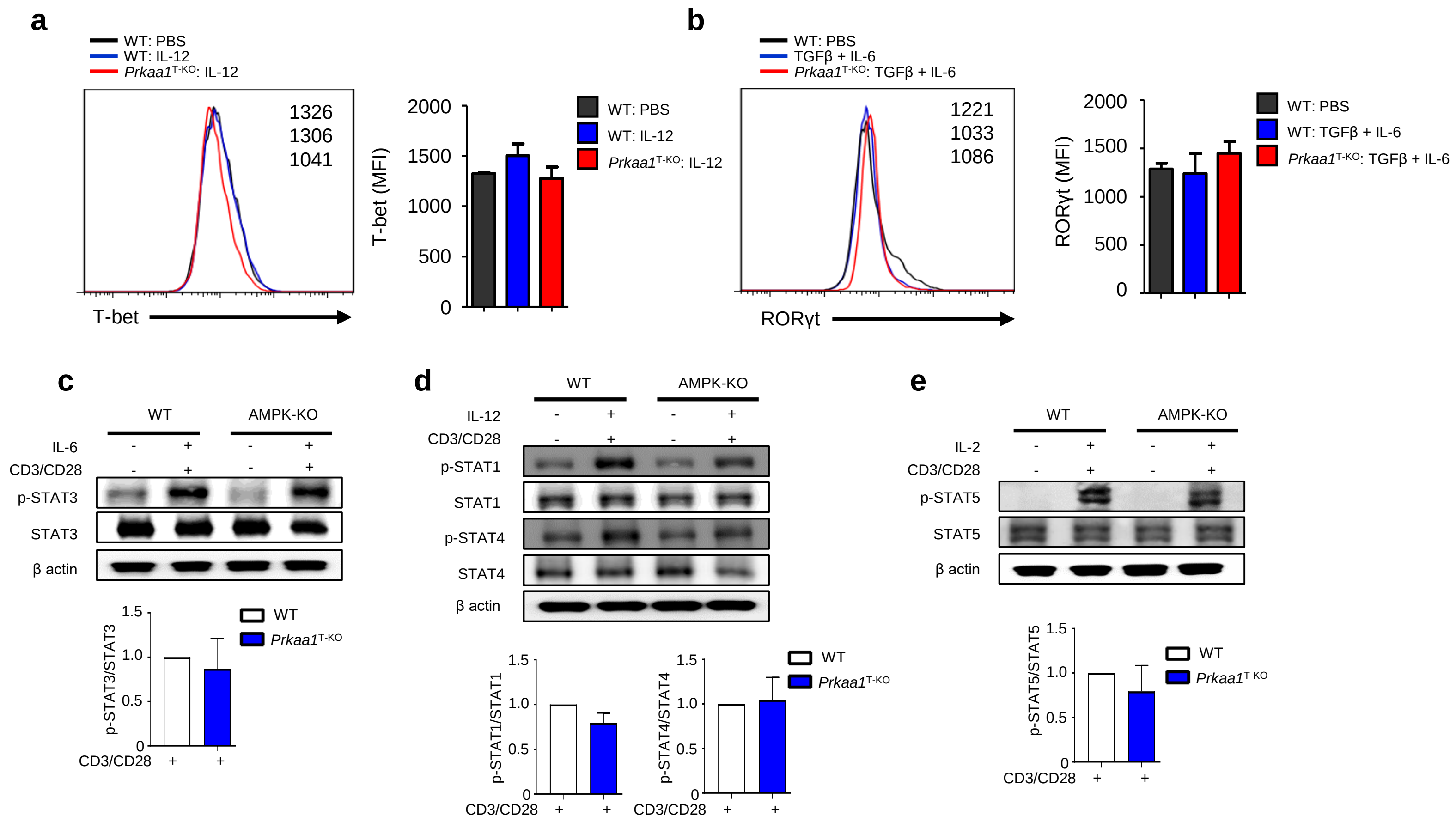

Supplementary Fig 2. *Prkaa1* deficient CD4⁺ T cell does not show altered Th1 and Th17: CD4⁺ T cell were sorted form WT and Prkaa1^{T-KO} mice and cultured in indicated conditions. **(a-b)** T-bet and RORγt expression under Th1 and Th17 skewing conditions. **(c-e)** Immunoblot analysis for total and phosphorylated (**c**) STAT3 (**d**) STAT1 and STAT4 (**e**) STAT5 were performed. β-actin as a loading control. The relative intensity and statistical analyses of TCR stimulated phospho-STAT proteins normalized to their respective total STATs bands were shown in bar graph. Data represents 3 independent experiments.

Supplementary Fig. 3

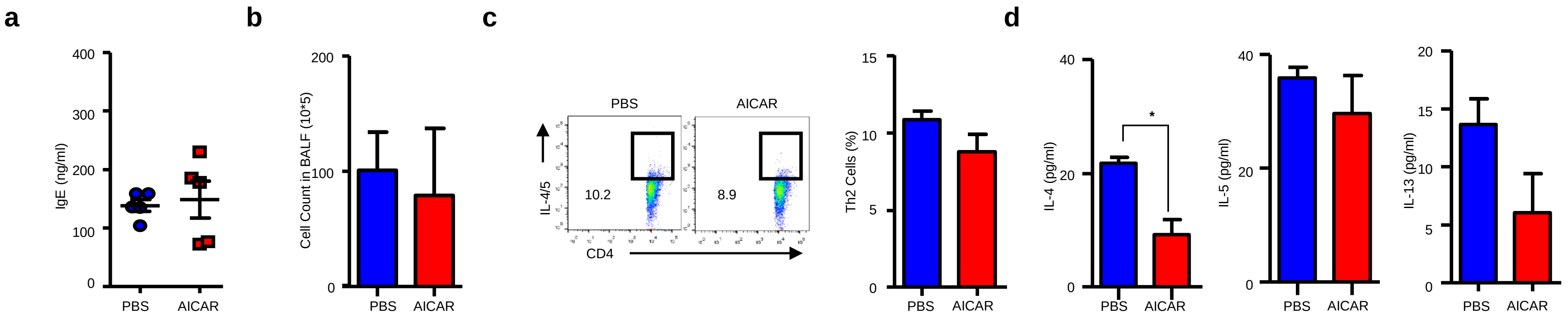

Supplementary Fig. 3 Allergic inflammation is weak to observe the effect of AICAR in C57BL/6 mice. **a** OVA-specific serum IgE level; **b** BALF cell count; **c** percentages of Th2 cells from mediastinal lymph nodes; **d** Th2 cytokine level in BALF. Data represent 3 independent experiments and are shown as mean ± SEM. $*P < 0.05$.

Supplementary Fig. 4

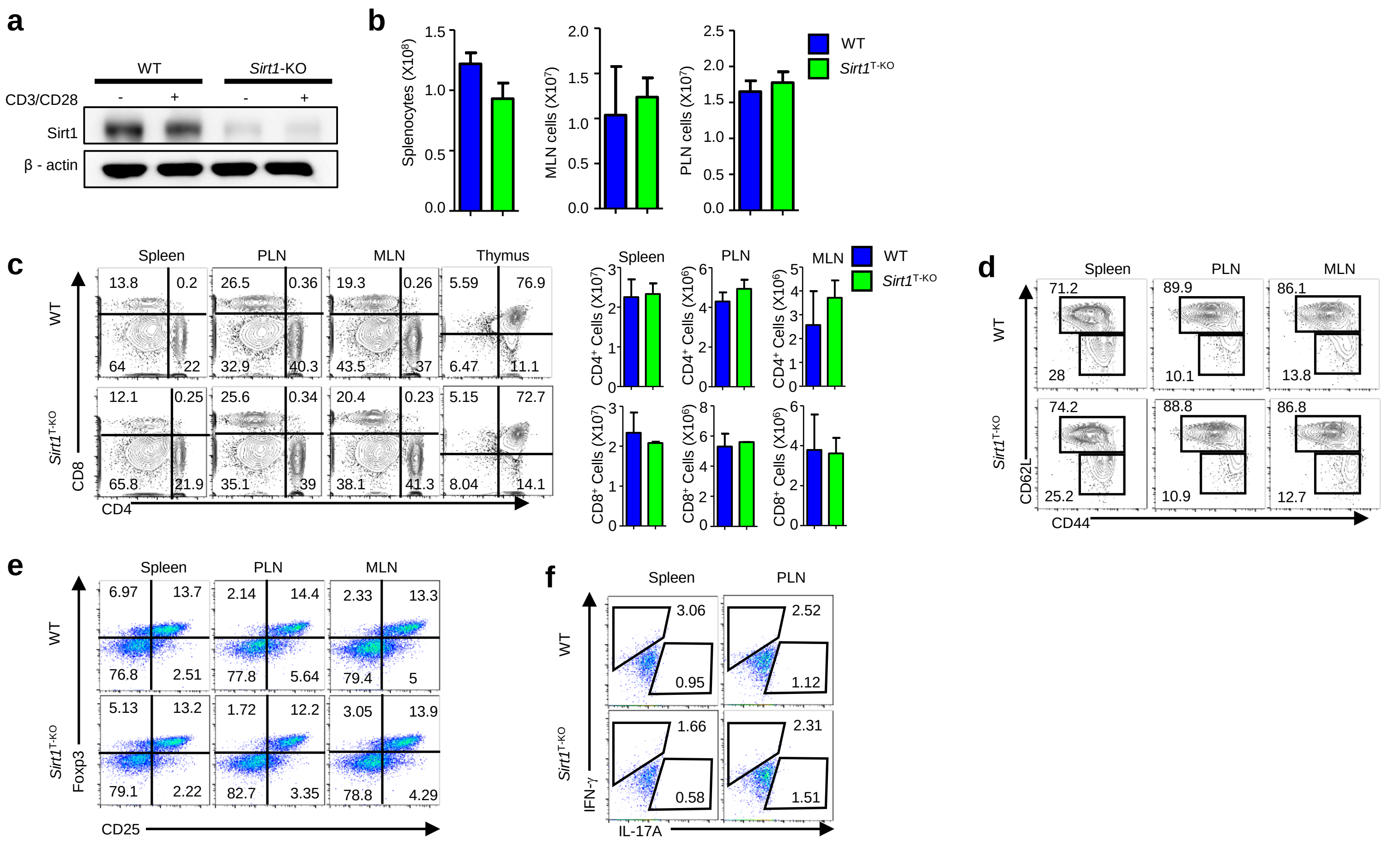

Supplementary Fig. 4 *Sirt1*$^{T\text{-}KO}$ mice show normal T cell phenotype. **a** Immunoblot analysis of SIRT1 protein expression in purified CD4$^+$ T cells from wild-type (WT) and *Sirt1*$^{T\text{-}KO}$ mice with/without TCR stimulation. β-actin was used as loading control. **b**–**f** Cells from spleen, peripheral and mesenteric lymph nodes (PLN and MLN), and thymus of 8-week-old WT and *Sirt1*$^{T\text{-}KO}$ mice. Cells were counted by hemocytometer and sub-populations of indicated tissues were analyzed by FACS. Total cell counts of splenocytes, MLN, and PLN (**b**), CD4 and CD8 frequencies and cell numbers on indicated lymphoid tissue (**c**), naïve (CD62L^{high}CD44^{low}) and effector memory (CD62L^{low}CD44^{hi}) CD4 T cells (**d**), CD25$^+$Foxp3$^+$ T_{reg} cells (**e**), and IFN-γ$^+$ (Th1) and IL-17A$^+$ (Th17) CD4$^+$ T cells (**f**). Results represent three independent experiments. Data are shown as mean ± SEM.

Supplementary Fig. 5

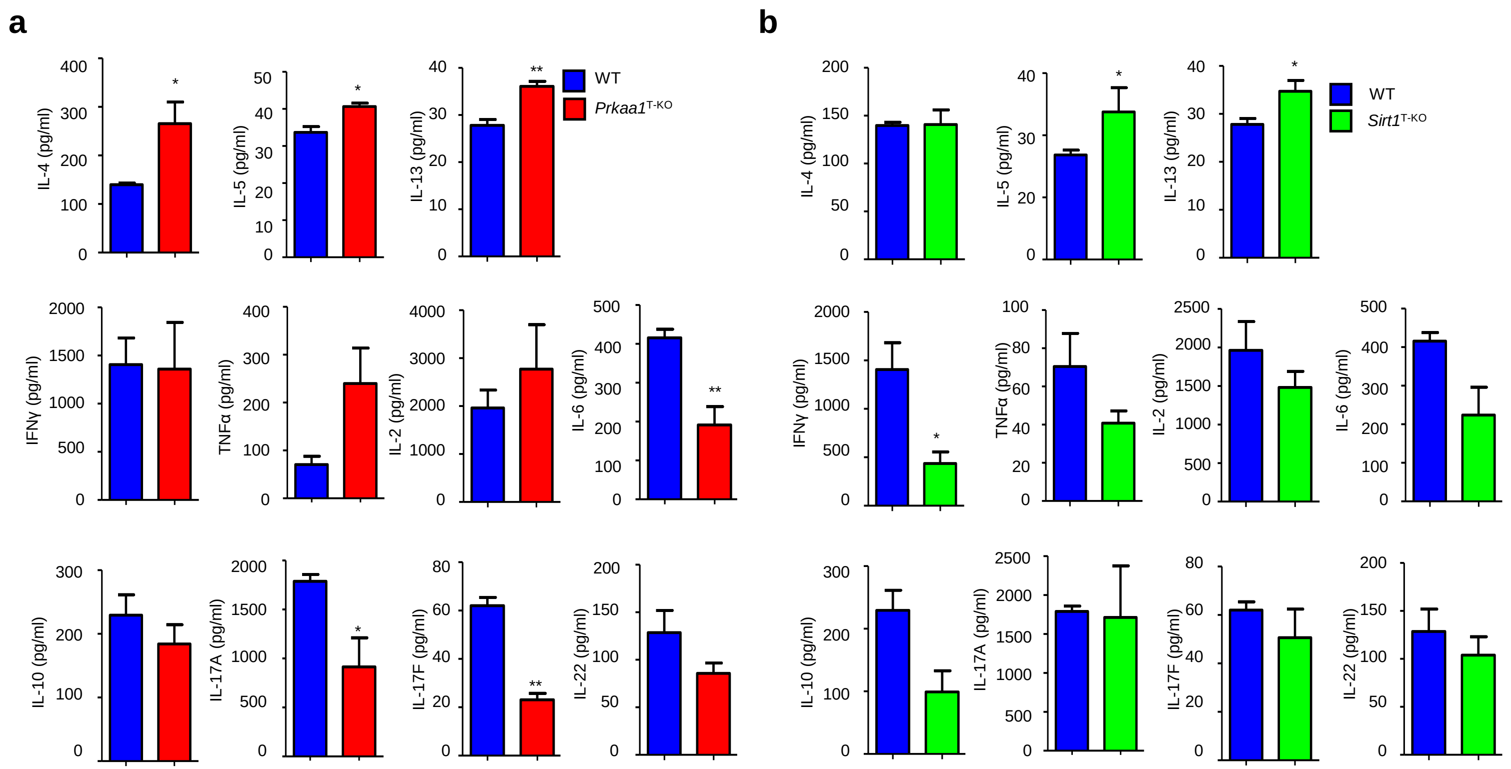

Supplementary Fig. 5 AMPK deficient and SIRT1 deficient CD4+ T cells exhibit similar cytokine profiles. **a** Pure CD4+ T cells were isolated from wild-type (WT) and *Prkaa1*$^{T\text{-}KO}$ mice. Cytokine bead array was done from isolated CD4+ T cells to evaluate different cytokine levels from *ex vivo* cultures. **b** Cytokine profiling was done to compare secreted cytokine levels from *ex vivo* cultures of CD4+ T cells from WT and *Sirt1*$^{T\text{-}KO}$ mice. Data represents 3 independent experiments and are shown as mean ± SEM. **P* < 0.05 and ***P* < 0.01

Supplementary Fig. 6

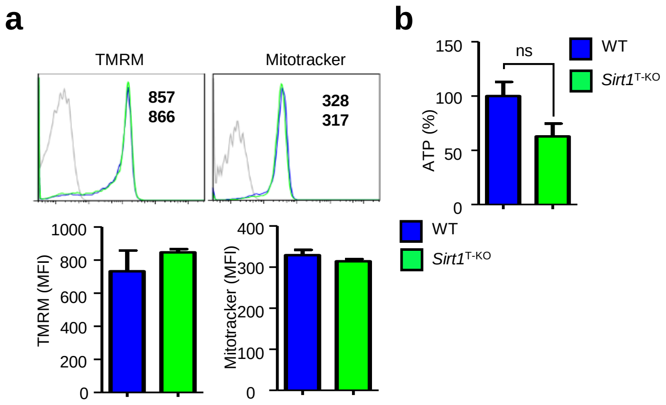

Supplementary Fig. 6 SIRT1 does not alter metabolic status of CD4$^+$ T cells. **a** Pure CD4$^+$ T cells were collected from wild-type (WT) and *Sirt1*$^{\text{T-KO}}$ mice. Tetramethylrhodamine methyl ester (TMRM) and Mitotracker were measured in WT and SIRT1-deficient CD4$^+$ T cells. **b** ATP production level was determined from WT and SIRT1-deficient CD4$^+$ T cells. Data represents 3 independent experiments and shown as mean ± SEM

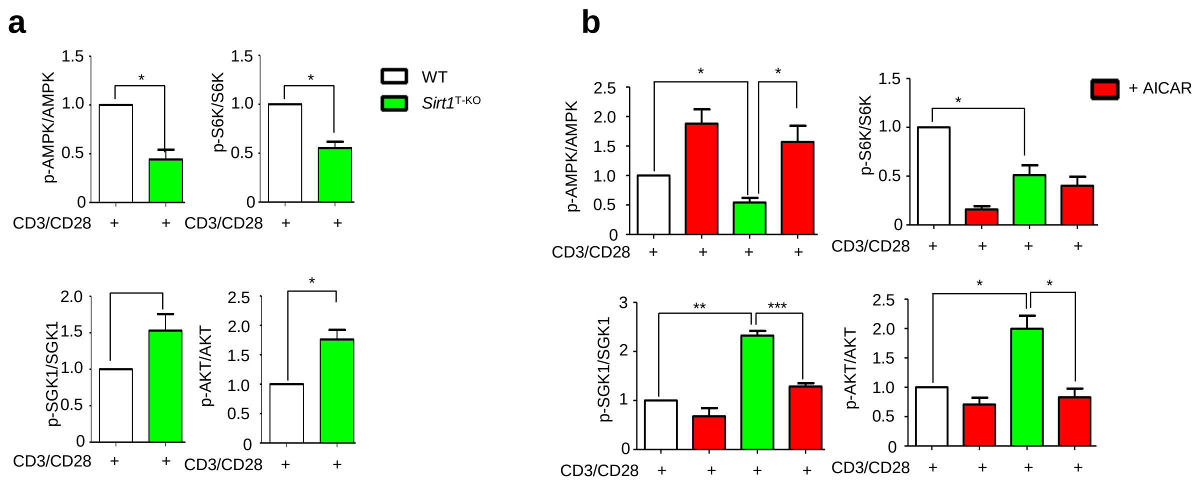

Supplementary Fig. 7 AMPK in CD4+ T cell regulates mTORC2 signaling. **a-b** Western blot of Fig 4A and 4B were quantified and shown in bar graphs **a** and **b** respectively. The relative intensities and statistical analyses of TCR stimulated WT and SIRT1 deficient CD4+ T cells. p-AMPK bands normalized to total AMPK, p-S6K normalized to S6K, p-AKT normalized to AKT and p-SGK1 normalized to SGK1 are shown in graphs. Data represents 3 independent experiments and shown as mean ± SEM. *P < 0.05 and **P < 0.01

Supplementary Fig. 8

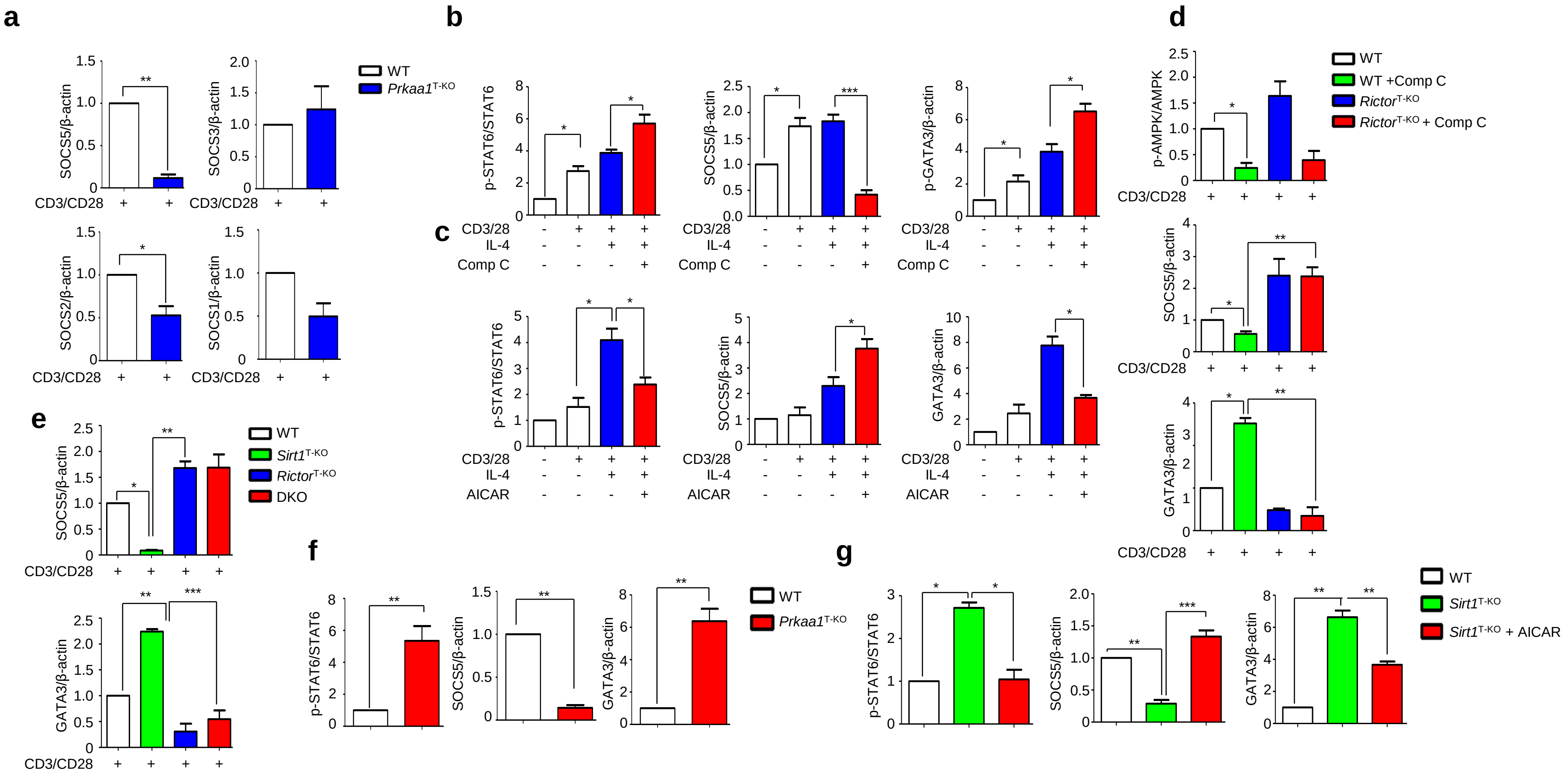

Supplementary Fig. 8 Activation of AMPK increases SOCS5 to suppress STAT6/GATA3 activity. **a** The relative intensities and statistical analyses of SOCS5, SOCS3, SOCS2 and SOCS1 bands of TCR stimulated WT and AMPK deficient CD4$^+$ T cells normalized to those of β-actin are shown as in Fig 6A, **b-c** Immunoblots as shown in Fig 6B and 6C were quantified and normalized expression of p-STAT6 to STAT6 and SOCS5 and GATA3 to β-actin are shown in graphs respectively. **d** TCR stimulated protein immunoblots of Fig 6D were quantified and normalized expression of p-AMPK to AMPK, SOCS5 and GATA3 to β-actin are shown in graph **e** Immunoblots as in Fig 6E from TCR stimulated were quantified and relative expression of SOCS5 and GATA3 after normalization to β-actin were shown in bar graphs. **f-g** The relative intensities and statistical analyses of immunoblot Fig 6F and 6G were calculated respectively. p-STAT6 band normalized to STAT6 and SOCS5 and GATA3 normalized to those β-actin are shown in bar graphs. Data represents 3 independent experiments and shown as mean ± SEM. *$P < 0.05$, **$P < 0.01$ and ***$P < 0.001$.
